# Closing the Loop on E‐waste: A Multidisciplinary Perspective

**DOI:** 10.1111/jiec.12645

**Published:** 2017-08-26

**Authors:** Ben Bridgens, Kersty Hobson, Debra Lilley, Jacquetta Lee, Janet L. Scott, Garrath T. Wilson

**Affiliations:** 1 School of Engineering at Newcastle University, Newcastle‐upon‐Tyne, NE1 7RU UK; 2 School of Geography and Planning at Cardiff University, Cardiff, UK; 3 Design School at Loughborough University, Loughborough, UK; 4 Centre for Environmental Strategy at the University of Surrey, Guildford, UK; 5 University of Bath, Bath, UK

**Keywords:** circular economy, industrial ecology, obsolescence, product lifetime, product service system (PSS), resource recovery

## Abstract

This paper describes the challenges faced, and opportunities identified, by a multidisciplinary team of researchers developing a novel closed loop system to recover valuable metals and reduce e‐waste, focusing on mobile phones as a case study. This multidisciplinary approach is contrasted with current top‐down approaches to making the transition to the circular economy (CE). The aim of the research presented here is to develop a product service system (PSS) that facilitates the recovery of valuable functional components and metals from mobile phone circuit boards. To create a holistic solution and limit unintended consequences, in addition to technological solutions, this paper considers appropriate component lifetimes; the (often ignored) role of the citizen in the circular economy; customer interaction with the PSS; environmental life cycle assessment; and social impacts of the proposed PSS. Development of enabling technologies and materials to facilitate recovery of components and metals and to provide an *emotionally durable* external enclosure is described. This research also highlights the importance of understanding *value* in the CE from a multifaceted and interdisciplinary perspective.

## Background

For the vast majority of human history “everything one owned was cherished, taken care of, and used to the very limits of its utility” (Fromm 2013, 61). In the decades following the World War II, the mechanization and automation of manufacturing in the Global North enabled the production and ownership of a wide range of consumer goods, to grow dramatically. The North American economy came to rely on continually increasing production, exemplified by the Ford Motor Company’s development of the assembly line, to provide the growth that is fundamental to the functioning of modern capitalist economies. As production started to exceed demand through the last century, the continuity of economic growth was threatened, and hence the *marketing men* were born. Working in sectors ranging from household goods to motorcars, they followed the lead of the clothing fashion industry where new styles drove purchasing long before products were worn out. To bring this trend into other sectors, one solution was *planned obsolescence* in its many forms—functional, aesthetic, psychological, and technological (London 2016; Packard 1963; Slade 2006; Cooper 2010). Where previously products were designed to be serviced and maintained for many years and often decades, the concept of an (increasingly short) *product lifetime* was created. The *throw away* culture had arrived and would expand to almost every type of product, spreading around the globe during the subsequent half century and still forming an integral part of global economies today (Cooper 2005).

Following this trend, and bringing us up to the modern day, mobile electronic devices such as tablets and smartphones are now frequently replaced before they cease to function, with the average first‐use life span of a mobile phone in the UK and United States being less than 2 years (Green Alliance 2016). Reasons for replacement include technological, cosmetic, or stylistic obsolescence, driven by manufacturers regularly releasing new models, each typically being slightly thinner and more powerful than previous versions. These design changes frequently result in devices that are more difficult to upgrade or maintain, a recent example being batteries that are glued into phones and tablets to save space, but which limit the functional life of the device to the life of the battery, often the only component with an inherently short life (Takeno et al. 2005).

Currently, very little electronic waste (e‐waste) is effectively reused at component level or recycled (Darby and Obara 2005; Suckling and Lee 2015). Instead, it is typically disposed of into landfill, incinerated, stored in a redundant state, or shipped to developing countries. In the United States in 2010, 141 million smartphones were discarded with 89% going to landfill (Green Alliance 2016). In 2009, estimates of global e‐waste varied from 20 million tonnes per year (Robinson 2009) to 40 million tonnes per year (UNEP 2016). Whichever figure is correct, these trends are causing severe negative environmental and social impacts due to the primary extraction of metals such as tantalum from coltan ore—essential for the functional components of electronic devices—along with e‐waste disposal and informal recycling in countries including China, Nigeria, Pakistan, and Ghana (Moran et al. 2014; UNEP 2016; Puckett et al. 2016, 2005; Luo et al 2011).

Research into the social consequences of e‐waste has examined its impacts on economies, cultures, and citizens’ health around the world. Kirby and Lora‐Wainwright’s (2015) detailed research into the practices and impacts of e‐waste disposal and recycling in China and Japan has underscored the vital role this resource has played in recent economic growth in East Asia. At the same time, the informal, unregulated, and insecure nature of this work exact significant negative health impacts on the many thousands in East Asia and beyond who are now reliant on this sector to generate a precarious living (see also Lora‐Wainwright 2016; Nnorom and Osibanjo 2008).

In response to the negative impacts of the linear *take‐make‐waste* economy, and its increasing fragility in the light of material scarcity and price volatility, there has been an increased focus on *closing the loop* on resources, exemplified in recent debates about the circular economy (CE) (e.g., Great Recovery 2016; EMF 2015; EC 2015; Braungart et al. 2007). Policy statements and programs framed around the CE from the European Commission (EC)—along with the work of the Ellen MacArthur Foundation (EMF)—primarily seek to influence designers and manufacturers to rethink how and why goods are created, used, and disposed of. In addition, there is an increasing awareness of the importance of engaging citizens in the CE, in terms of consumer acceptance of new *models of consumption* (Edbring et al. 2015) and wider questions about the social and cultural consequences of the proposed circular production‐consumption systems (Hobson 2016).

In response to the above challenges, the “Closed Loop Emotionally Valuable E‐waste Recovery” (CLEVER) research project was developed at a research “sandpit” organized by the UK Engineering and Physical Sciences Research Council, which brought together academics from a wide range of disciplines to develop creative solutions to increase *resource efficiency*. The CLEVER team combines expertise across several disciplines (chemistry, material science, engineering, life cycle analysis, social science, and product design) to propose a route to achieving circular material flows in consumer electronics, taking into account technical, economic, social, and environmental barriers and opportunities. In this paper, we present a product service system (PSS) that could facilitate this transition, describe the challenges, and opportunities that the development of this closed loop system has identified and present key developments that have arisen from addressing these challenges.

## Understanding “Value” in the Circular Economy

The CE is normally described in terms of circular flows of materials, being: “a simple, but convincing, strategy, which aims at reducing both input of virgin materials and output of wastes…” (Haas et al. 2015, 765). However, it can equally be seen as a way of maintaining the value of products, components, and materials. One approach advocated by proponents of the CE is *design for longevity* (Great Recovery 2016; Park 2016). Whether increasing product longevity minimizes environmental impacts depends on the balance between impacts at the various stages of the product lifetime, and the end‐of‐life (EoL) strategy (Kwak and Kim 2012; Cooper 2010). As such, “Sustainable manufacturing requires products to be developed with a predetermined useful life, which will minimise resource usage and environmental impact based on their intended End‐of‐Life strategy*”* (Kara et al. 2008, 1). For mobile phones, materials extraction and manufacturing account for an average of 74% of lifetime carbon dioxide emissions (Suckling and Lee 2015). It follows that to utilize resources more efficiently and reduce e‐waste, consumers could be encouraged to retain their devices for longer and return them at the end of their functional life (Cooper 1994; Van Nes et al. 2016; Chalkley et al. 2016; Wilhelm 2012). However, encouraging people to keep products for longer is notoriously difficult due to a combination of perceived technological obsolescence, social status, and superficial damage to the product. These factors are all strongly driven by marketing strategies from companies that rely on rapid turnover of these devices, particularly as sales growth slows in the increasingly saturated smartphone market. Yet, circular component and material flows could significantly reduce materials and manufacturing costs (EMF 2016), offsetting the negative economic consequences of reduced sales.

A more sophisticated approach is to consider the optimal lifetime and “value” of *each component*, rather than treating the device as a single object (Kara et al. 2008; Ji et al. 2013). A performance economy approach, where the objective is to maintain the quality of stock rather than focusing on flows of components or materials through the economy, is an approach that facilitates this view (Lee et al. 2016). For example, is the optimum value realized by reusing the phone, or by capturing and reusing the components? What are the optimal timings for interventions in the product life cycle? This analysis of the value of stock can inform the development of a PSS, as interventions (such as upgrading components or replacing a complete unit) require the active participation of the owner or user. This approach also requires consideration and understanding of expected component lifetimes at the design stage to ensure components that need to be replaced can be readily accessed when required, and that they are designed with the appropriate level of durability. The viability of repair and upgrade are determined by a combination of factors: the costs of carrying out the process; the overall cost of the product; the current cost of replacement parts; and, crucially, the desire (or not) of the owner to upgrade components or to purchase a new model. Costs will vary through the life of the product, but the designer can greatly increase the likelihood of component replacement by designing for disassembly (Harjula et al. 1996) and by designing for emotional durability (Chapman 2015).

The potential economic benefits of the CE to manufacturers are clear, in particular by reducing reliance on volatile global material supply chains (EMF 2013). Research has recently drawn attention to the importance of cultural and social values in the transition to the CE, both in terms of enabling an effective transition, and in ensuring that the CE is beneficial to consumers. For example, the uptake of various PSSs has been shown to depend on, and sometimes clash with, personal preferences and shared norms (e.g., cleanliness, convenience, and novelty) when consumers are required to alter their current consumption patterns, for example, hiring rather than purchasing equipment (e.g., Edbring et al. 2015; Catulli 2012). As such, getting consumers “on board” with revamped business models will arguably be a more substantial challenge than some high‐profile commentary on the CE suggests (Hobson 2016; Hobson and Lynch 2016).

This point signals a larger issue with current policy and governance approaches to fostering a CE. For the most part, the role of the citizen has been assumed to be that of a rather passive consumer, accepting (or not) a new PSS (Hobson and Lynch 2016), with the main focus being on *designing in* and then retrieving value postuse via new business models. However, decades of research into efforts to promote *sustainable lifestyles* in the Global North has underscored the need to explicitly question the values at play around current consumption levels and practices, rather than attempt to “hide” sustainability in products and services (Hobson 2013). That is, while we can make business models that, on their own, create greater material circularity, the potential for significant *rebound* effects (e.g., Figge et al. 2014) across broader consumption patterns remains very real, as consumers remain unengaged and uninformed about the very issues that the CE aims to address (Hobson and Lynch 2016). In short, the CE needs to be considered as much as a social and political project, as it does a business, scientific, and technological endeavor.

## A Product Service System to Close the Loop on E‐waste

**Figure 1:**
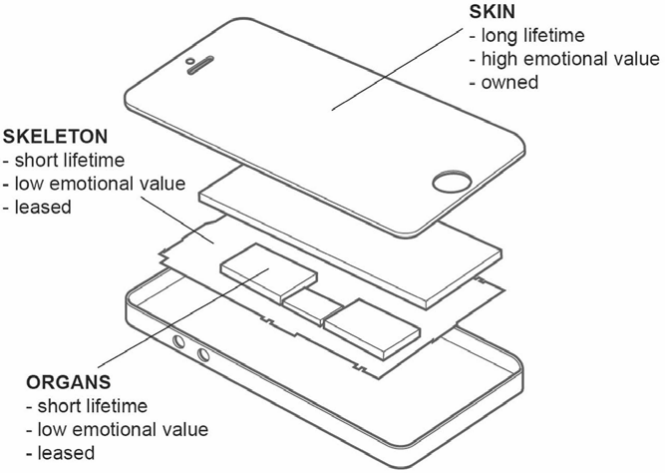
Functional and emotional value of different parts of a mobile phone is suggested by conceptualizing them as the skin, skeleton, and organs. The lifetime of components may be dictated by loss of performance and technological advances (in the case of the skeleton and organs) or by accidental damage and/or cosmetic obsolescence (when considering the skin).

Mobile electronic devices, in particular smartphones, provide a challenging, but potentially high‐impact, case study for transitioning to a CE. To give the scale of the current problem context, it is estimated that there are between 28 and 125 million unused mobile phones in UK homes (Green Alliance 2016). According to a recent survey of 181 18‐ to 25‐year‐old mobile phone owners (Wilson et al. 2017), each mobile phone was owned by a participant for just under 5 years, of which the phone spent just under 3 years unused in storage. The materials contained within these “hibernating” devices are effectively lost, thereby necessitating increased production and the harvesting of raw materials to meet market demand. Additionally, the value contained within these phones diminishes over time, as illustrated by figure 1; therefore, their recovery is time‐sensitive (Wilson et al. 2017). To keep materials in a *closed loop*, enable the recovery and recycling of valuable resources, and reduce premature disposal and resulting sustainability impacts, consumers must be encouraged to return their devices such that the functional components can be retained in the manufacturing loop.

Several recent developments in mobile phone design have tried to address issues of material provenance, ethical sourcing, and social impact (e.g., Fairphone) as well as modular design for ease of upgradability (e.g., Phonebloks/Project Ara, PuzzlePhone, and nexpaq).^1^ These innovative and thought‐provoking projects each address one aspect of the problems associated with mobile phone manufacture and disposal, but do not provide a complete solution. Fairphone—“the smartphone with social values”—focuses on accountability and reduction of negative impacts in the supply chain and social impacts of manufacturing, with some consideration of EoL. While the work on supply chains and manufacturing has provided a powerful demonstration that complex supply chains can be audited and managed, there is little focus on ensuring that valuable metals remain in the manufacturing loop at EoL. The modular Project Ara (formerly Phonebloks), PuzzlePhone, and nexpaq provide potential for personalization and component upgrade without replacing the whole product. However, there is currently no information about how modular components would be recovered, and, if they were, whether they can easily return to the manufacturing loop. Additionally, the potentially high turnover of modular components could result in increased e‐waste, and would arguably discourage attachment to the device, reinforcing the notion that a mobile device’s value resides ostensibly in content and functionality rather than physical form (Wiberg 2016).

In contrast to these examples, the CLEVER project has developed a hypothetical PSS for reduction of e‐waste which considers all aspects of the product life cycle, with a particular focus on facilitating recovery of components and return of materials to the manufacturing loop, as these aspects tend to be lacking in other projects. A PSS is a function‐oriented business model incorporating a mix of both service and ownership, offering a viable method for reducing material consumption by *“*shifting the business focus from designing (and selling) physical products only, to designing (and selling) a system of products and services which are jointly capable of fulfilling specific client demands*”* (Manzini and Vezzoli 2003, 851).

The starting point for the design of the CLEVER PSS was the acknowledgement that mobile phones are comprised of multiple components, each with different longevity requirements, value‐chain lifetimes, and recovery and disposal/recycling routes, engendering a need for different levels of consumer awareness and engagement (Wilson et al. 2015). Therefore, we started by conceptualizing the device as comprising of three parts: a *skin*—the outer casing, or the part that the user interacts with directly; a *skeleton*—the critical support components inside the device (e.g., circuit board); and *organs*—the electronics that deliver the product’s core functionality (e.g., processor and other electronic components) (figure 1). Rather than viewing the mobile phone as a single value entity, in terms of both emotional and functional value, we have separated out the components to facilitate the recovery of subassemblies and components when it is more *appropriate* to do so.

Taking this concept of a mobile phone as a carrier of components with disparate lifetimes, the CLEVER project developed a PSS to serve as a case of excellence; to map out the offer and how the user interacts with the offer; to establish the actors in the system; and to consider the environmental, social, and economic advantages of that system (Vezzoli et al. 2016). The hypothetical PSS was developed from a designer‐centric perspective, created through a “top down” workshop involving CLEVER investigators and researchers. The collective expertise of the participants can be described thusly: material testing, characterization, and material aging; social and environmental transformation, and multilevel environmental governance; social life cycle assessment (LCA) approaches, and social planetary boundaries; renewable raw materials, particularly cellulose, for the development of functional materials; and user‐centered sustainable design approaches to generate behavioral insights to drive design development of less‐resource intensive products. The workshop was facilitated by a sustainable design expert, with prior experience in service and PSS design. The objectives for the workshop were that the PSS must achieve a closed loop, enabling the optimum flow and efficient recovery of valuable materials and maximizing the remanufacture and reuse of components where possible; the PSS should avoid technocratic solutions to offer the user a sense of empowerment and engagement while also providing tangible advantages relative to other current models, such as data security and back‐up options (physical and virtual); and the PSS should take into account and deal with social, economic, and environmental impacts while avoiding negative rebound effects. Although the PSS was intended as a hypothetical exemplar rather than a specific business proposition, the PSS had to have a “sense” of commercial viability by being able to demonstrate scalability, and provide a steady and predictable (re)manufacture stream that illustrates value retention beyond first use.

The primary workshop methodology was *business origami*, a creative and generative methodology primarily used in the early phases of a design process with multidisciplinary teams to paper‐prototype a system model (Hanington and Martin 2016). Two‐dimensional paper tokens representing the system elements were maneuvered around a table by the project team with paper arrows added and constantly readjusted in the light of project‐wide discussions to represent the interactions between these elements. During the workshop, tokens were used to represent factories, countries, transport (freight, lorry, and domestic), *skeletons* and *organs*, groups of people, buildings and infrastructure, mobile phones, *skins*, and the user. Post‐It notes were used for annotating the emerging system and to capture arising questions. Once complete, the environmental and social hotspots were mapped and then the service proposition refined with the above objectives in mind.

**Figure 2:**
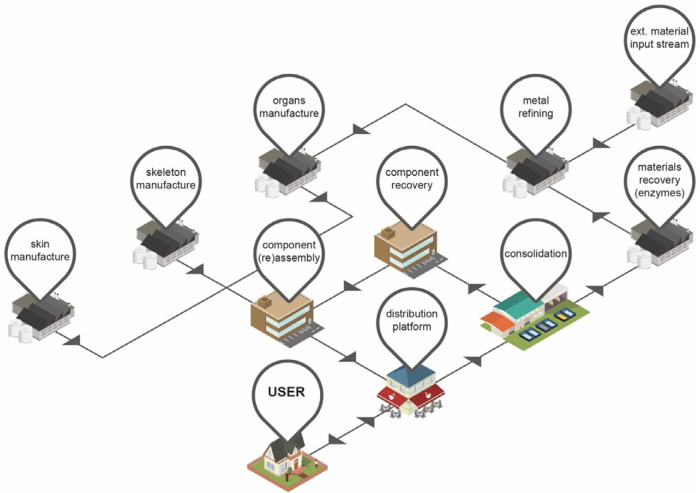
An overview of the proposed “Closed Loop Emotionally Valuable E‐waste Recovery” Product Service System.

From the workshop, the emphasis of the proposed PSS (figure 2) shifted toward upgrading the hardware in use as opposed to replacing the complete phone. This suggests different ownership models and lifetimes for the different components. The CLEVER PSS was designed as a hybrid of *use‐oriented* (for *skeleton* and *organs*) where ownership of the tangible product is retained by the service provider, who leases the functions of the product, and *product‐oriented* (for the *skin*) where ownership of the tangible product is transferred to the consumer, but additional services, such as maintenance, may be provided (Tukker 2004). The *skeleton* and *organs*, which have low‐emotional value but require regular technical upgrades, are owned by the service provider and leased to the customer. They are designed with the shortest initial life, but with the focus on ensuring that they are recovered for subsequent reuse, remanufacturing, or, at worst, targeted materials recovery. The service provider will be well‐placed to facilitate more effective management of a staged recovery process for valuable components and metals at the optimal point of value recovery (Lee et al. 2016; Suckling and Lee 2015). The *skin* component, which would be owned by the customer, is designed to accrue emotional “value” and stimulate the desire to maintain it and return the device at strategic points for refurbishment and internal upgrade of the *skeleton* and *organs*. Heirloom *skin* materials have been engineered to reveal variations in surface appearance over time and were designed to create intrigue or surprise (Ludden et al. 2012) and stimulate ongoing interaction. By challenging contemporary notions of everlasting perfection (e.g., Maffei and Fisher 2013) and acknowledging the passing of time and acquisition of experiences and memories within the surface *patina* of the object (Giaccardi et al. 2014; Odom and Pierce 2016; Chapman 2009; Rognoli and Karana 2014; Pedgley 2016), it was envisaged that the device would be “endowed with greater significance by and for its user” (Turner and Turner 2013, 404) as the more self‐expressive value the product acquires the stronger the emotional bond becomes (Chapman 2009; Lacey 2009; Mugge et al. 2005; Van Nes and Cramer 2006; Mugge et al. 2009).

It could be argued that it may be more beneficial (and more straightforward) to design a PSS in which both the skin and the functional components are recovered and replaced. However, a pure leasing model negates the potential for citizen engagement in the CE and reduces their role to that of a passive consumer (Hobson and Lynch 2016). Additionally, without feedback on cause and effect, consumers may be less likely to learn from, and adapt, their behavior accordingly (Lilley and Wilson 2013). It is also suggested that objects which are leased rather than owned receive a lower level of care. A study of car‐sharing (Bardhi and Eckhardt 2012) identified that Zipcar customers are not motivated to appropriate the cars and do not feel a sense of ownership. This, in turn, contributes to a diminished sense of obligation for object stewardship: “I’ll parallel a Zipcar in a tighter spot than I would with mine because it’s not mine. I’m just not worried about it” (Bardhi and Eckhardt 2012, 9).

## How Would the Product Service System Work?

In the proposed PSS, the user would purchase the service contract and associated phone from a wide range of possible distribution platforms, including e‐commerce and online stores, traditional bricks‐and‐mortar retail and dedicated upgrade centers, local franchises (based within, e.g., a local coffee shop), independent community upgrade shops, or vending machines. Over time, the external enclosure of the device changes gradually and becomes personalized, giving the owner a reason to keep the device rather than replace the whole unit. When an internal upgrade is required (or desired), to mitigate technological obsolescence, the user returns the phone via one of many possible distribution platforms and the phone receives internal hardware and software upgrades as requested. For the upgrade process to be acceptable to the consumer, particularly given the “feelings of panic and anxiety” (Vincent 2006, 39) that some people experience when separated from their mobile phone, a rapid replacement of the *organs* is required—perhaps in as short a time as it takes to drink a cup of coffee. The phone is then returned to the user with the new internal components and the user retains the valued external *skin* of the phone. The reclaimed components from the individual platforms are consolidated for sorting. After consolidation, *organ* and *skeleton* components and subassemblies are either recovered for reassembly and reuse, or sent for material recovery and metal refining. Mixed metal recycling streams can input into the metal refining process here, maximizing the use of external resources to negate any process losses. Raw materials from the materials recovery and metal refining processes are remanufactured into new *organ* and *skeleton* components. Recovered and new components and subassemblies are assembled (as hardware upgrades, or as new phone assemblies with new *skins*) and circulated to the distribution platforms in order for the circular process to be repeated (figure 2).

## Discussion

There are multiple challenges and opportunities inherent in operationalizing the PSS described above, which span a range of disciplines and highlight the collaborative approach that will be required to successfully effect a wider transition to the Circular Economy.

### Enabling Technology: Materials Which Age Spectacularly

To ensure that phones are returned for upgrade of the functional *organs* (as opposed to disposal or “hibernation” when a new model is purchased), our approach is to engender an emotional connection between the external enclosure (*skin*) of the phone and the user. This approach required close collaboration between a product designer and materials engineer to test and develop materials, and understand the experiential design requirements and carry out user testing.

Natural materials, including wood, leather and stone, and some metals, are commonly described as “aging gracefully” and develop a *patina* which is valued more highly than the new material (Odom and Pierce 2016; Candy et al. 2016; Pye 1968). Examples include *verdigris*—the durable, green surface finish of weathered copper, which is highly valued as a building cladding material, and complex changes in wood due to weathering, which tend to emphasize both the visual appearance and texture of the grain (Domone and Illston 2010; Hoadley 2016). In stark contrast, man‐made materials can elicit strong negative emotions when they are no longer new: “… disgust for degraded, evidently used, worn, no longer pristine plastic items that may invite their disposal” (Fisher 2004, 30). The product context must be considered, and consumer electronics “tend to occupy a synthetic and scratch‐free world of slick polymers…” (Chapman 2014, 141) with wear or damage to the pristine exterior contributing to the rapid replacement of these devices.

To assess the validity of using natural materials which “age gracefully”’ in the context of consumer electronics, a user study was carried out which explored tactile and aesthetic responses to new and artificially aged mobile phone cases made from bamboo, walnut, cork, leather, brushed titanium, plastic, and rubber (Bridgens et al. 2015; Lilley et al. 2016). While there were some positive responses to the aesthetic changes of natural materials (bamboo and walnut) to simulated aging, the most popular materials were durable man‐made materials with a smooth, shiny surface, with titanium being the favorite. Preferences are strongly influenced by the materials that people expect to see in a particular context: for mobile phones, people expect sleek, uniform materials and it was generally felt that natural materials were out of place.

This provided a clear design requirement for the *skin*: to develop a material surface that was initially smooth and uniform to meet consumers’ expectations for the surface of a new product, yet would provide novelty, personalization, and graceful change with use. To develop such a material requires an understanding of how people physically interact with (handle, carry, and store) the product to allow prototype materials to be tested using accelerated wear testing, before embarking on long‐term (time‐consuming and expensive) user testing. Product testing of electronic devices by manufacturers typically focuses on avoidance of functional failure, not gradual wear and longevity, and there are no published methods or standards for accelerated wear testing for this type of product. While there are a small number of studies about use habits and people’s proximity to their phones (Dey et al. 2016; Deloitte 2016; Van Laerhoven et al. 2016), there is no published information about wear and damage of these products in use. This highlights an important barrier to design for longevity and circularity—existing design processes and test methods for consumer products focus on the pristine object at point of sale, not how that object will change over time.

Test methods were therefore developed based on observed typical use and handling. The broad spectrum of possible degradation mechanisms (Manley et al. 2015) were divided into two main types of interaction:

1. *Wear*: analogous to careful use and handling, and carrying in a pocket or case, which gradually polishes the material over time. Existing studies of the coefficient of friction and roughness of human skin, and contact pressure when objects are held (Chen et al. 2009; Derler et al. 2009; Tomlinson et al. 2009; Derler and Gerhardt 2011; Skedung et al. 2011; Wongsriruksa et al. 2012; Kim et al. 2013), enabled a suitable material to be found which is tribologically similar to human skin (known as *Lorica*), which could be used to polish test materials with a known contact pressure.
2. *Damage*: to simulate less careful use and storage, such as carrying the phone in a pocket with keys, or dropping onto a rough surface. This test is based in manufacturers’ durability testing (link to video: www.youtube.com/watch?v=HicdXV_47V8). The mobile phone is fixed to the side of an inclined rotating cylinder, and a selection of keys and coins are placed in the cylinder. The number of revolutions of the cylinder is used to control the severity of the damage.

**Figure 3:**
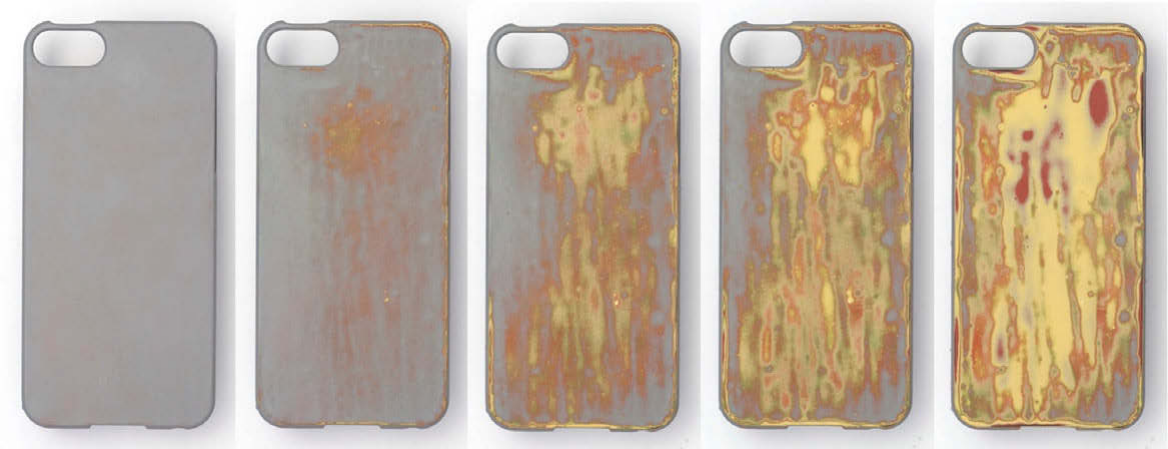
Simulated aging of a mobile phone case with layered surface coating, which transitions from a uniform surface to meet consumer expectations of a new product, and then “ages spectacularly” to provide surprise and personalization through use. The example shown has been artificially worn using emery paper and is not a result of user testing.

A layered surface finish was chosen to provide an initially uniform exterior that would age “spectacularly”—gradually wearing with use to reveal colored layers, in unique patterns that reflect the user’s interactions with the phone. The accelerated aging methods described above, combined with adhesion testing to ASTM D 3359 with a cross‐hatch adhesion tester, allowed candidate coatings to be tested to ensure that they were strong enough and sufficiently well adhered to not chip or crack when dropped or scratched, but fragile enough that they would gradually wear away and reveal the layers beneath in use (figure 3).

**Figure 4:**
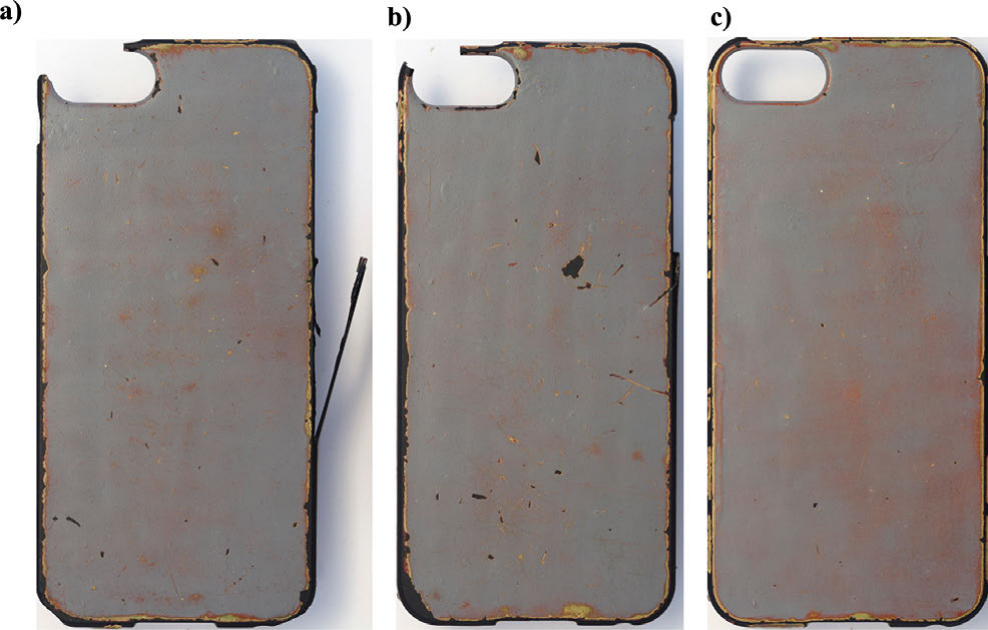
Example of mobile phone cases with layered surface coating after 4 months’ use. Instead of the anticipated gradual wear to the material surface, the cases have chipped and scratched (a and b). (c) Is starting to reveal patterns from gradual wear, but the surface has chipped on the angular edges of the Apple i‐phone 4.

A user study with thirty‐six 18‐ to 25‐year‐old participants was undertaken to investigate people’s response to the layered surface coating as it changed in use. The layered surface was applied to *Apple i‐phone 4* cases, which could be placed over participants’ own phones, allowing them to interact as usual with their phone. Twelve participants were given cases with no coating (control sample), 12 received coated cases with no pre‐explanation of the potential for material change to occur, and 12 were explicitly informed that the coated cases they were given “*had the potential to change*.” The study was set up to run for 6 months, allowing the effect of context and acclimatization to gradual change to be studied, as opposed to the visceral response in many materials studies where participants are presented with material samples (Lilley et al. 2016; Wongsriruksa et al. 2012). The intention was to interview participants and photograph the phone cases at 2, 4, and 6 months. However, after 4 months it was clear that the layered surface was not changing as intended, and instead was chipping and flaking, and that damage to the plastic case was also occurring (figure 4).

The damage to the layered phone surface showed that the accelerated aging test methods do not reflect the actual “wear and tear” that occurs to a mobile phone in use. There is a clear lack of knowledge of how users interact with their possessions, which will hinder the development of materials that are designed to age or change in particular ways to increase product longevity.

At the 4‐month point, the study was stopped, and the participants were shown images and video of the simulated ageing of the layered case (similar to figure 3). These elicited a generally positive, but mixed, response with comments including: “*It would be cool to keep using it and it would be really cool to use that cover*.”; “*I think that would have been quite cool actually… that would look like a phone case design I would maybe buy as it looks quite cool*”; and “*I wouldn’t have wanted it. It’s too ugly. There are too many dull colours mixed together*.” This suggests that the proposed strategy of using materials which “age spectacularly” to engender emotional attachment may be viable, given further development of the layered material surface.

### Enabling Technology: Materials for Triggered Degradation

Implementation of the PSS requires devices that can be readily disassembled to facilitate upgrading and, as ease of disassembly is also key to component recovery and recycling, many *design for disassembly* strategies have been developed (Li et al. 2015). In this context, *active disassembly* relying on shape memory polymers or alloys (Chiodo and Jones 2012; Chiodo et al. 2002) is particularly attractive, but even devices designed for disassembly are usually not reduced to their individual component parts, but instead to groups of components combined into functional units.

The circuit board is a case in point: Once separated from the other functional units, this may be submitted to current recycling processes that entail pyrolysis and production of metal slags, followed by refining and electro‐winning processes to recover the more valuable metals with a particular focus on gold (Hagelüken and Corti 2010). Currently, printed circuit boards are manufactured from very robust epoxy resins, glass fiber, and layers of conductive metals. Recycling tends to be focused on recovery of valuable metals, usually employing destructive methods such as pyrolysis followed by copper recovery, typically by the “black copper smelting method” (Ghodrat et al. 2016) and hydrometallurgical leaching processes. Traditional and newer technologies have been reviewed (Zhang and Xu 2016), but most remain predicated on the need for high‐energy methods for destruction of the circuit board support elements, although greener technologies, such as comminution and flotation processes, are gaining popularity (Estrada‐Ruiz et al. 2016). Opportunities for improved metal recovery and optimized separation processes arise if circuit boards can undergo *triggered degradation* releasing individual components and metals contained in contacts (usually gold) and circuits (including gold and silver). Triggered disassembly is well known in nature, where materials that are robust during their useful lifetimes are readily (bio)degraded at EoL.

In CLEVER, a nature‐inspired approach was adopted and cellulose, a key structural component of plant cell walls and the largest volume renewable biopolymer known, was selected as the basis for new *skeleton* materials. While cellulose is highly insoluble, eons of evolution have provided many organisms with enzymes capable of degrading cellulose into its constituent sugars, which are consumed as fuel. Cellulose alone is unlikely to have the properties needed in electronic component support materials, or circuit boards, but cellulose based composite materials may be engineered with key characteristics such as stiffness, fire retardancy, water (non)absorption, and surface adhesion/printability.

**Figure 5:**
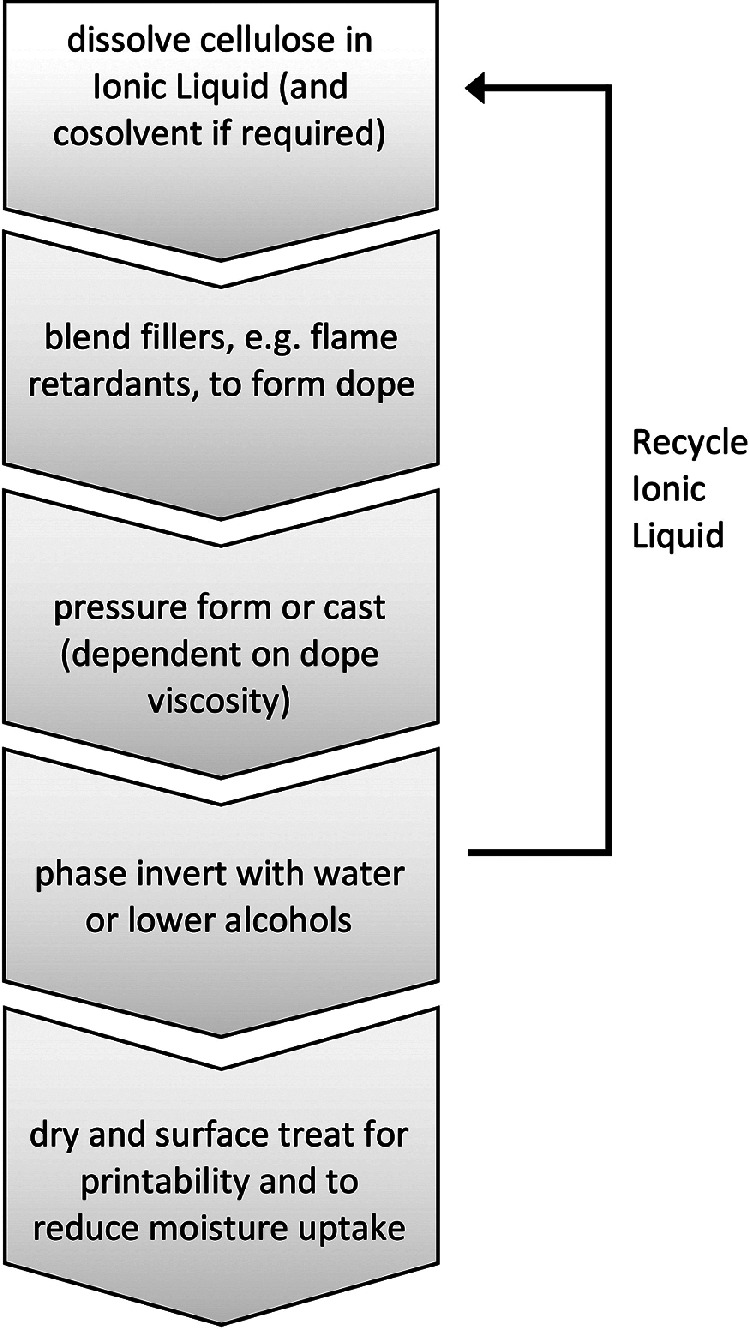
Summary of the process used to manufacture cellulose‐based “skeleton” support materials. The ionic liquid of choice is 1‐ethyl‐3‐methylimidazolium acetate, selected as the most efficient and least hazardous ionic liquid for dissolution of cellulose. As this processing aid is costly, it must be recycled.

**Figure 6:**
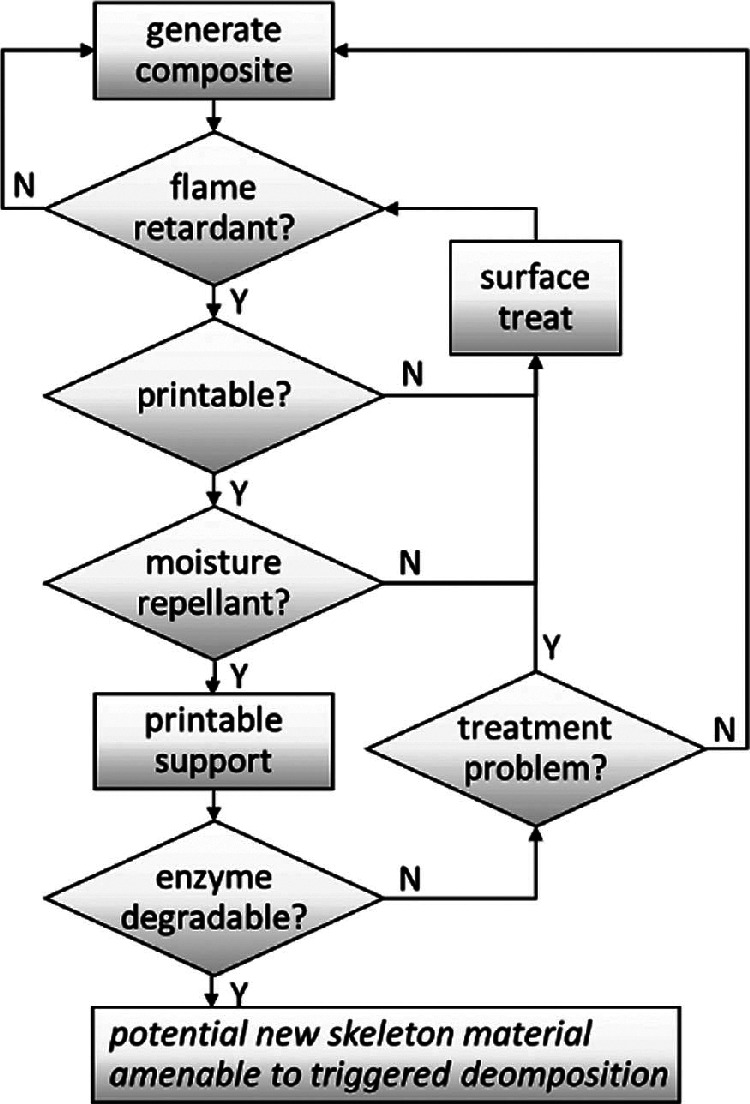
Simplified flow chart showing the skeleton materials development process with multiple iterative steps.

The materials tested were prepared using ionic liquid mediated dissolution with a range of co‐solvents (Gale et al. 2016) and regeneration of cellulose following the method of Rogers and coworkers (Swatloski et al. 2002) with various blended fillers and surface treatments (figure 5). Generating composites with appropriate characteristics required a series of iterative processes of material production, characterization, and testing,

Figure 6. Finally, after many iterations, a composite of cellulose with ammonium polyphosphate filler (10% by weight), surface treated with ethylcyanoacrylate to generate a very thin (<0.1% by weight) moisture impermeable polymer layer on the surface was arrived at (Chandrasekaran et al. 2016).

As the triggered degradation step was intended to be achieved using commercially available enzyme cocktails, testing of enzymatic degradation of the materials was also required. The second‐generation biofuel industry has driven rapid development in cellulose saccharification enzyme preparations (Kuhad et al. 2016), thus these are available at reasonable cost. Using enzymatic degradation of circuit boards and other supports allows use of very mild conditions and enables potentially impactful recovery and recycling opportunities: (1) Recovery of individual small components, such as transistors, is enabled, and (2) extension of the concept of materials designed for triggered degradation to flexible circuitry and other “connecting” components could reduce losses of valuable metals during “preprocessing” (shredding and separating) (Chancerel et al. 2009).

To gain an understanding of where the most significant deleterious environmental impacts might arise during the production of a new *skeleton* material, an environmental analysis was conducted based on data derived from laboratory‐scale materials preparation. This hotspot analysis was designed to provide information that could be acted upon early in the development process. It does not replace a full LCA, but highlights processes/materials that must be carefully considered and possibly altered, or replaced during development. Environmental impacts associated with energy use were identified as the greatest impacts, but, as laboratory‐scale processes are not optimized in this regard, this is unlikely to reflect impacts in a manufacturing process. The next greatest impact arose from production of the purified cellulose raw material, suggesting that use of less rigorously purified celluloses would be desirable. This is relatively simple to address, as much of the impact in cellulose production results from processes designed to yield bright white material, and residual color in the skeleton materials is acceptable. The process of composite production itself was designed to facilitate recovery and recycling of virtually all solvents and processing aids.

### Assessment of Social and Environmental Impacts

Throughout the PSS development process, the environmental and social implications of the new materials and systems have been considered and used as a decision‐making tool. This required collaboration between all disciplines involved in the project—development work by a product designer, materials engineer, and chemist—was iteratively assessed by experts in LCA and social science to ensure the proposed systems were truly beneficial.

Streamlined scoping LCA‐type studies that have been utilized as full environmental LCA (ELCA) studies are both time‐consuming and contain considerable uncertainty, especially where products and systems are not yet fully defined. While these scoping studies have been based on agreed standards (ISO 2006), their benefit lies more in creating a better understanding of the systems under investigation, rather than the calculation of specific impacts. In essence, they provide signposts to issues within the life cycle that warrant further investigation. A combination of value chain analysis (where value is not limited to economic value), alongside an ELCA‐type study, can be useful in highlighting how best to develop a PSS such that the maximum benefits are obtained for the minimum negative impact.

A further challenge is the evaluation of the potential social and economic impacts of altered PSSs. In recent years, various methods have been developed and tested for undertaking *social* LCAs (see Macombe et al. [2011] for a review), including for existing electronic products such as laptops (Ekener‐Petersen and Finnveden 2013). This work has highlighted the benefits of considering, for example, the labor conditions and broader social benefits for those working in the manufacture of electronic products. However, this work remains contentious given the issues of collecting meaningful and rigorous data on social impacts at such work sites, as well as knowing which social and economic categories reflect the greatest impact hotspots. For hypothetical and proposed PSSs, the challenges of evaluating potential social and economic impacts are even greater and require further research effort if the CE is to promote social as well as environmental sustainability (Hobson and Lynch 2015; Jørgensen 2013). For example, if the informal e‐waste recycling centers in China are eradicated through a new PSS that keeps recycling in the Global North, will the costs to the Chinese workers’ livelihoods be outweighed by their potential improvement in health outcomes?

Addressing the above question is both an issue of how one weighs different evaluative criteria and the scale of implementation of any new or proposed PSS. Here, scale suggests both the market share captured by a new PSS, as well as the geographical scope encompassed within a “closed loop,” for example, keeping materials and processes within the European Union (EU), as recent policy documents suggest (e.g., EC 2011). In addition, the scale of implementation of technical processes (i.e., chemical processing, manufacturing, and material recovery) affects their efficiency and other impacts, while the scale of adoption of a particular technology, style, or brand will affect its social acceptability and popularity with different groups of people. Thus, for a proposed PSS, there are often too many hypotheticals and contingencies to undertake meaningful social impact analysis.

## Conclusions

It is widely accepted that a transition from the current *take‐make‐waste* economy, which has been ubiquitous since the industrial revolution, to the CE will be beneficial both in terms of reducing environmental impacts and improving the profitability and stability of manufacturing operations. However, the wider social implications of this transition are poorly understood, difficult to measure, and, to date, have not been considered by policy makers and CE advocates as key to a successful transition.

Even more challenging than assessing the impacts of the CE is how to effect this transition. Technical challenges must be overcome to reverse decades of industrial design that gave minimal consideration to disassembly, repair, component reuse, or material separation and recovery. Current approaches to the CE are typically “top‐down” and are led by designers and manufacturers and focus on these technical challenges. However, understanding the interaction of the citizen‐consumer with the CE is vital to ensure willing participation and to avoid rebound and unintended consequences.

We have proposed a PSS which facilitates e‐waste recovery by engaging the consumer with the product they own and interact with, rather than by financial coercion. Technological obsolescence is mitigated by upgrade of the functional components while cosmetic or stylistic obsolescence is mitigated by engendering emotional attachment between the owner and the exterior of the device. Note that in the proposed PSS, it is the external enclosure (which has negligible monetary value and limited environmental impact) that is designed to be the most enduring component. The aim here is not to reduce the need to manufacture external enclosures, but to engender an emotional bond with the user.

A broad multidisciplinary approach is required to enable an effective, beneficial transition to the CE. For the example discussed here, a team with expertise in chemistry, material science, engineering, LCA, social science, and product design were required, and worked closely together to ensure a coherent outcome. This level of cross‐discipline collaboration is still uncommon and often challenging. Time is required to build working relationships and achieve effective communication and productive collaboration—suggesting that long‐term research grants or industry collaborations are required to achieve novel circular systems to deliver the products that consumers take for granted with significantly reduced impacts.

## Footnotes

1 Fairphone www.fairphone.com; Project Ara https://atap.google.com/ara/; PuzzlePhone www.puzzlephone.com; nexpaq https://nexpaq.com/intro/, all accessed September 2016.
